# Hepatocyte Growth Factor-Loaded Biomaterials for Mesenchymal Stem Cell Recruitment

**DOI:** 10.1155/2013/892065

**Published:** 2013-06-18

**Authors:** Julia van de Kamp, Willi Jahnen-Dechent, Bjoern Rath, Ruth Knuechel, Sabine Neuss

**Affiliations:** ^1^Institute of Pathology, Aachen University Hospital, RWTH Aachen University, 52074 Aachen, Germany; ^2^Biointerface Group, Helmholtz Institute of Biomedical Engineering, Biointerface Group, RWTH Aachen University, 52074 Aachen, Germany; ^3^Department of Orthopaedic Surgery, Aachen University Hospital, RWTH Aachen, 52074 Aachen, Germany

## Abstract

Human adult mesenchymal stem cells (MSC) can be readily harvested from bone marrow through aspiration. MSC are involved in tissue regeneration and repair, particularly in wound healing. Due to their high self-renewal capacity and excellent differentiation potential *in vitro*, MSC are ideally suited for regenerative medicine. The complex interactions of MSC with their environment and their influence on the molecular and functional levels are widely studied but not completely understood. MSC secrete, for example, hepatocyte growth factor (HGF), whose concentration is enhanced in wounded areas and which is shown to act as a chemoattractant for MSC. We produced HGF-loaded biomaterials based on collagen and fibrin gels to develop a recruitment system for endogenous MSC to improve wound healing. Here, we report that HGF incorporated into collagen or fibrin gels leads to enhanced and directed MSC migration *in vitro*. HGF-loaded biomaterials might be potentially used as *in vivo* wound dressings to recruit endogenous MSC from tissue-specific niches towards the wounded area. This novel approach may help to reduce costly multistep procedures of cell isolation, *in vitro* culture, and transplantation usually used in tissue engineering.

## 1. Introduction

Wound healing is involved in all processes of tissue regeneration and repair. Its complex processes depend on the proper interactions between cells of different origin and extracellular matrix (ECM) components. Beside, cells of the immune system and diverse resident cells, mesenchymal stem cells (MSC) play a key role in wound healing [1].

Human MSC can be isolated from various tissues (e.g., bone marrow or adipose tissue) and their stem cell characteristics are described in detail since the pioneering work of Friedenstein and coworkers in 1968 [2, 3]. In addition, MSC possess immunomodulatory and trophic properties, making them a promising cell source for regenerative medicine [4]. Endogenous MSC migrate towards the damaged area, participating strongly in the wound healing response through paracrine communication [5]. Paracrine communication occurs via a concerted action of bioactive factors, such as vascular endothelial growth factor, epidermal growth factor, keratinocyte growth factor, and hepatocyte growth factor (HGF) [6, 7]. HGF is outstanding because of its proangiogenic and chemotactic properties. Its receptor c-met is expressed in MSC, making the cells migrate towards a higher HGF gradient. This gradient is additionally established by macrophages and apoptotic cells [8, 9]. Furthermore, MSC participate in the wound response through the secretion of ECM molecules, thus influencing tissue remodeling and wound contraction [10].

In chronic wounds, healing processes are disordered and delayed requiring medical intervention to improve the healing situation [11]. Biomaterials can serve as wound dressings to provide a structure in case of severe tissue loss. Natural and artificial biomaterials can influence cell behavior in different ways, for example, in viability, proliferation, and differentiation. Among the wide range of prospective biomaterials, fibrin and collagen are suitable candidates for wound healing [12, 13]. Fibrin, a native component highly involved in blood coagulation, is a key factor in wound healing as shown in one of the first animal studies [14]. Over the years, fibrin has come to use as a tool for cell and drug delivery [15–17] as well as for the expansion of cord blood-derived hematopoietic stem cells [18]. The first use of collagen as a natural matrix has enhanced wound healing in a porcine study [19]. In recent years, collagen has been shown to induce chemotaxis and haptotaxis of rabbit and human MSC [20]. It also exhibits increasing valuable influence on the interaction and differentiation of MSC [21]. Collagen is a potential nonimmunogenic degradable scaffold for the incorporation of MSC in dermal tissue engineering [10].

In the present study, we analyzed the effect of HGF incorporated into fibrin and collagen gels on the recruitment and migration of MSC using standardized *in vitro* assays. We carried out a scratch assay, a simple, time-, and cost-efficient method to evaluate cell migration [22]. In addition, we used a modified Boyden chamber assay to evaluate the chemotactic activity of HGF on cell migration [23].

In summary, the concept of MSC being involved in the process of wound healing and tissue repair is widely accepted. Fibrin, collagen, and HGF are useful components to direct MSC migration *in vitro* and *in vivo*. Our study sheds light on the motility of MSC in the context of two degradable biomaterials in combination with HGF. We describe for the first time a robust and directed MSC migration towards HGF released from fibrin and collagen *in vitro*. These results pave the way for the development of a recruitment system for endogenous MSC to improve wound healing in burns or chronic diseases *in vivo*.

## 2. Materials and Methods

### 2.1. Mesenchymal Stem Cell Isolation and Cell Culture

Procedures were approved by the local ethics committee. Human donors gave informed consent. Human mesenchymal stem cells (MSC) were isolated from femoral heads according to the protocols of Haynesworth et al. and Pittenger et al. [3, 24]. Briefly, femoral heads of patients undergoing total hip joint endoprosthesis were rinsed several times with stem cell medium, containing 60% DMEM, 40% MCDB-201, 1x ITS + BSA-linoleic acid, 1 nM dexamethasone, 100 *μ*M ascorbic acid, 10 ng/mL EGF, 40.000 U Penicillin, 40 mg Streptomycin, and 2% FCS (PAN Biotech, Aidenbach, Germany). Cell suspension was transferred to a 50 mL tube and centrifuged at 500 g for 10 minutes. The cell pellet was resuspended in fresh medium and seeded in a T75 tissue culture flask. After 24 hours, nonadherent cells were removed by medium change. At 80–90% confluence, stem cells were trypsinized with stem cell trypsin (Invitrogen, Darmstadt, Germany) and reseeded in a density of 5.000 cells/cm^2^ for optimal proliferation. Medium change occurred every 3-4 days. All cells were characterized by flow cytometry and multipotency using standard protocols as required by the International Society for Cellular Therapy [25, 26] and as previously described [8]. Cells were incubated in a 20% O_2_ and 5% CO_2_ humidified atmosphere at 37°C. Cells in passages between 2 and 5 were used for the experiments.

### 2.2. Fibrin Gel Preparation

Fibrin gels were prepared by polymerization using thrombin and fibrinogen as described before [27]. A fibrinogen solution was prepared by dissolving 160 mg fibrinogen powder in 8 mL of water (*aqua ad iniectabilia)* in 8 mL GBSH5 buffer incomplete. The mixture was transferred into a dialysis tube and equilibrated with GBSH5 buffer incomplete at 4°C over night. The next day, the fibrinogen solution was placed into an Oak Ridge Centrifuge Tube and centrifuged at 1200 g for 30 min. The clear supernatant was aspirated, sterile filtrated, and frozen at −80°C. Directly before cell seeding, CaCl_2_ buffer (50 mM in ddH20, sterile filtrated) and GBSH5 buffer complete were added to fibrinogen. For polymerization, 10 *μ*L thrombin (1000 U/mL) and 180 *μ*L fibrinogen solutions were mixed in a 24-well plate. In addition, 75 ng/mL HGF was added before polymerization. The plate was shaken carefully to enhance polymerization and incubated at 37°C for 20 min. After polymerization, medium was added. All results were based on five independent experiments (*n* = 5).

### 2.3. Collagen Gel Preparation

Collagen gels were generated as previously described [28, 29]. Briefly, eight volumes of acidic collagen G (3 mg/mL collagen I/III in 12 mM HCl) were mixed with one volume 10-fold DMEM (both from Biochrom, Berlin, Germany), following neutralization with 2 M sodium hydroxide. One volume of medium ±75 ng/mL HGF (PromoCell, Heidelberg, Germany) was added to the gel mixture. The gel was placed in the bottom compartment of a 24-well plate and incubated at 37°C for 2 h to polymerize, and medium was added afterwards. All results were based on five independent experiments (*n* = 5).

### 2.4. HGF ELISA

HGF was incorporated before polymerization of the gels in a 96-well plate. Supernatant was collected after 1, 2, 4, 6, 8, 24, 48, and 168 hours of culture. HGF ELISA (PromoCell GmbH, Heidelberg) was carried out according to the manufacturers' instructions. Briefly, standards and samples were incubated in a 96-well plate at 4°C overnight. The next day, biotin antibody was added to each well and incubated for 1 h at room temperature. Afterwards, streptavidin solution was added and the plate was left to incubate for 45 minutes at room temperature. Next, TMB one-step substrate reagent was added to each well and incubated for 30 minutes at room temperature. Stop solution was added before the plate was read at 450 nm.

### 2.5. Scratch Assay

**Figure 1:**
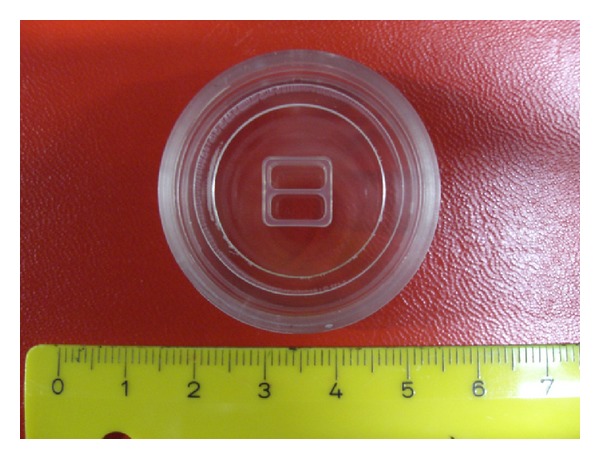
Culture-insert in a 24-well format dish to produce uniform scratches in parallel samples.

Cells were grown until confluence in both compartments of a culture-insert (ibidi GmbH, Martinsried, Germany) inside a 24-well format dish (Figure 1). The inserts were used for creating uniform scratch dimensions throughout the experiments. Inserts were removed carefully and medium ±75 ng/mL HGF was added to each well. Closure of the resulting *in vitro* wound was documented photographically at 0, 8, 16, and 24 h after removal of the insert. Pictures were processed with Adobe Photoshop 7.0 and analyzed with Octave 3.2.4.

### 2.6. Boyden Chamber Assay

For analysis of directed cell migration, MSC were seeded in a density of 10^5^ cells/mL in the top compartment of a transwell system (Corning Costar, Corning, USA) (modified Boyden chamber) in a 24-well plate. The top compartment was separated from the bottom compartment by a polycarbonate membrane with 8 *μ*m pores. To exclude a solely chemokinetic effect of HGF on the migration of MSC, we simultaneously added the same concentration of HGF to the top and the bottom compartment and also only to the top compartment. Collagen or fibrin ±75 ng/mL HGF was deposited in the bottom compartment as described above. This assay was termed “migration assay.”

To mimic the *in vivo* situation and to check for possible delayed release of HGF from the biomaterials, collagen and fibrin were both coated with a thin layer of Matrigel (BD biosciences, Franklin Lakes, USA). This assay was termed “invasion assay.” To analyze any chemotactic/chemokinetic influence of Matrigel itself on the cells, the bottom of the lower compartment of the transwell (without biomaterials) was covered with a thin layer of Matrigel.

In both assays, basal migration (no HGF, no biomaterial) was used for normalizing values; medium +HGF in lower compartment served as an internal control. Cells were allowed to migrate for 24 hours before analysis and quantification. After removal of the transwell, the top cell layer was wiped off with a lint-free cloth (Wepa, Arnsberg, Germany); membranes were fixated and stained with Hemacolor (Merck, Darmstadt, Germany) and mounted on objective slides with cover slips and Vitro-Clud (Langenbrinck, Emmendingen, Germany). Cells on the bottom side of the membrane were quantified in 5 different high-power fields per membrane at 200-fold magnification. Each analysis was performed in triplicate for five different donors.

### 2.7. Statistical Analysis

Data were presented as mean values ± standard deviation (SD). One-way ANOVA and Tukey's posttest were used for statistical analysis; significance was defined as *P < 0.05, **P < 0.005, ***P < 0.001. The corresponding graphical representations were generated with GraphPad Prism 4.0 (GraphPad Software Inc., San Diego, USA).

## 3. Results & Discussion

### 3.1. HGF ELISA

**Figure 2:**
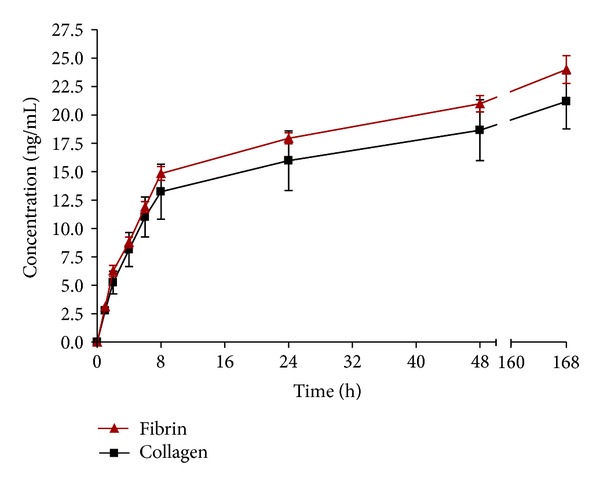
HGF (ng/mL) released from fibrin and collagen gels over time. 75 ng/mL HGF was incorporated into collagen and fibrin gels. Supernatant was used for ELISA and gels without HGF served as control. *n* = 2.

The release kinetics of HGF from fibrin and collagen gels was analyzed with a human HGF ELISA kit. During the first 8 hours, HGF was released quickly both from collagen and fibrin (17% and 20%, resp.). After this time, HGF was released in a more gradual way. In total, both biomaterials show a comparable release kinetic. However, a higher concentration of HGF was flushed out from fibrin compared to collagen. After a total of 168 hours, the cumulative releases of HGF from collagen and fibrin were about 28% and 32%, respectively (Figure 2). A continued gradual release process of HGF is expected, because over 60% of HGF is still present in the biomaterials. After the initially strong release, the favored slow release of HGF is expected, which is reasonable for *in vivo* applications. Therefore, our system shows promising results *in vitro.* In our ongoing *in vivo* study (mouse), we specifically focus on the inflammatory phase, in which several growth factors, such as platelet-derived growth factor and vascular endothelial growth factor, are released rapidly by macrophages to attract inflammatory cells [30]. Our HGF kinetics can be correlated to the physiological conditions described by Clark and Henson [31] as well as Cohen and colleagues [32].

Xu and colleagues studied the controlled release of HGF from a bovine scaffold for vocal fold reconstruction. They came to similar release kinetics for HGF from acellular scaffolds. They showed a total release of 32.6% HGF after 7 days, which is in accordance with our results [33]. For both of our gels, the HGF release is well acceptable and will be analyzed further in terms of cell migration and degradation properties in our *in vivo* model.

A study on the potential of collagenous matrices as release carriers of exogenous growth factors was carried out by Kanematsu et al. [34]. They analyzed the *in vivo* release of several growth factors (bFGF, HGF, PDGF-BB, VEGF, and HB-EGF) from a collagen sponge over a period of 28 days. After 7 days of implantation, 70% HGF was released from the matrix and a strong correlation between the release profile and the degradation profile of the matrix was found.

### 3.2. Scratch Assay

**Figure 3:**
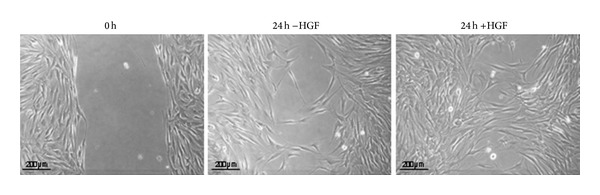
MSC ±HGF after 0 and 24 hours. All assays were carried out with 4 different donors in passages 2 to 5. One representative donor is shown exemplarily. Scale bars: 200 *μ*m.

**Figure 4:**
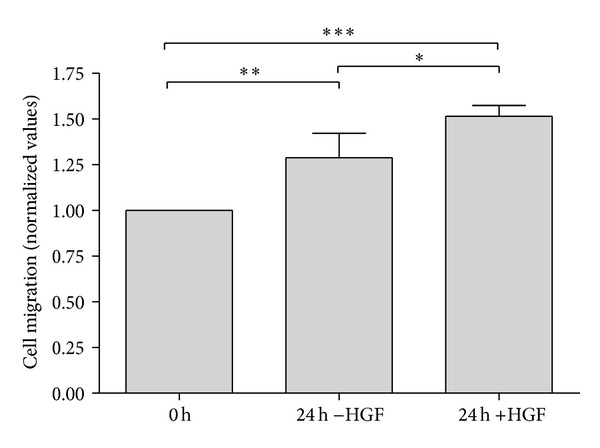
Analysis of HGF-dependent MSC migration after 24 hours in the presence and absence of HGF. Results based on *n* = 4. *P < 0.05, **P < 0.01, ***P < 0.001.

Based on a scratch assay by Neuss et al. [8], a cell migration assay was carried out (Figure 3). Neuss and colleagues determined the HGF concentration which was optimal for cell migration in the range of 50 to 100 ng/mL. We chose 75 ng/mL for all assays. MSC were cultured in culture inserts until confluence; inserts were removed (=0 hours) and stem cell medium ±75 ng/mL HGF was added. No difference in cell migration in the presence or absence of HGF was observed after 8 and 16 hours (data not shown). However, after 24 hours, MSC cultured with HGF showed a higher number of migratory cells (approx. 50% of cell-free area covered) than the counterparts without HGF (approx. 25% of cell-free area covered; Figure 4).

A number of *in vitro* studies reported on HGF as a chemoattractant for different cell types, thus underlining our findings. All studies showed an enhanced, if not significantly increased, cell migration in the presence of HGF compared to counterparts cultured with less concentration of HGF or no HGF [35–38]. Interestingly, cotreatment of HGF with transforming growth factor-*β*1 led to superior cell migration than HGF alone, suggesting a combination of cytokines as beneficial for wound healing, too [39]. Recently, a new quantitative approach for analyzing long-term kinetics of wound healing was introduced. Adenocarcinoma cell lines migrated in a coordinated and collective fashion and expressed a higher cell velocity in the presence of HGF, therefore showing accelerated wound closure [40]. In summary, HGF has a strong chemotactic influence on the cell migration of different cell types.

### 3.3. Boyden Chamber Assay

**Figure 5:**
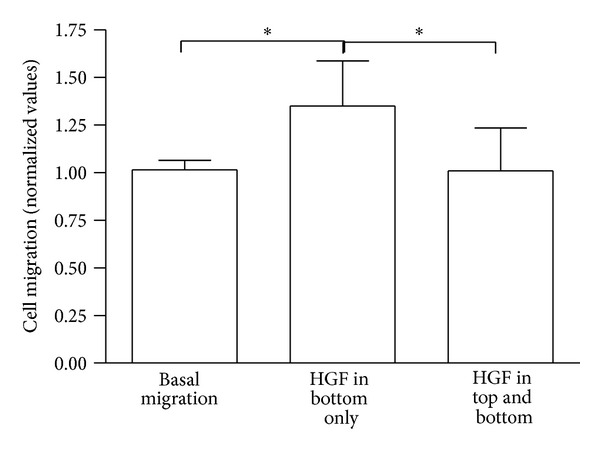
Boyden chamber assay with HGF in top and bottom compartment. All values compared to basal migration and based on *n* = 4. *P < 0.05.

**Figure 6:**
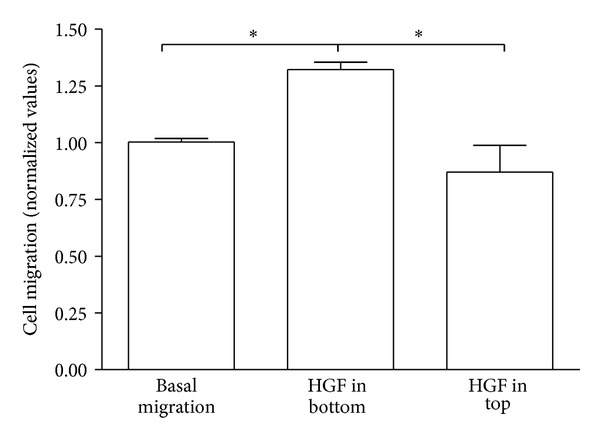
Boyden chamber assay with HGF in top compartment only. Values compared to basal migration and HGF in bottom compartment only. *n* = 2. *P < 0.05.

To further evaluate the migratory influence of HGF on MSC, we based our first Boyden chamber experiment on the results described by Neuss and coworkers [8]. To check for any chemokinetic influences of HGF, the assay was carried out with the same concentration of HGF but differently localized (no HGF = basal migration, bottom compartment only, top and bottom compartment, Figure 5; and top compartment only, Figure 6). The chemotactic effect of HGF is significantly stronger than the chemokinetic effect. MSC migration is clearly enhanced (*P < 0.05; Figure 5) when compared to basal migration and when equal amounts of HGF are present in both compartments simultaneously. This effect has also been described by Zheng et al. [41] and confirms our hypothesis of the chemotactic effect of HGF on the migratory behavior of MSC.

**Figure 7:**
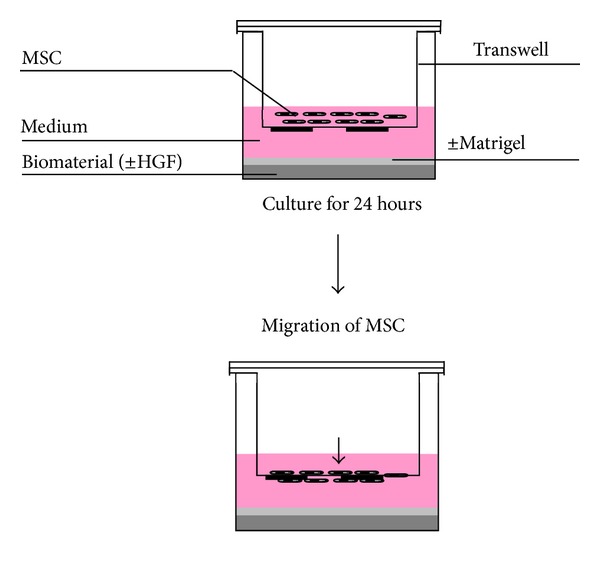
Sketch of modified Boyden chamber assay.

Based on the scratch assays results, we further investigated the effects of HGF incorporated into collagen and fibrin using a modified Boyden chamber assay. For both assays, migration (biomaterials) and invasion (biomaterials plus a thin layer of Matrigel), collagen and fibrin were either free of or loaded with 75 ng/mL HGF (Figure 7).

*Migration Assay*

**Figure 8:**
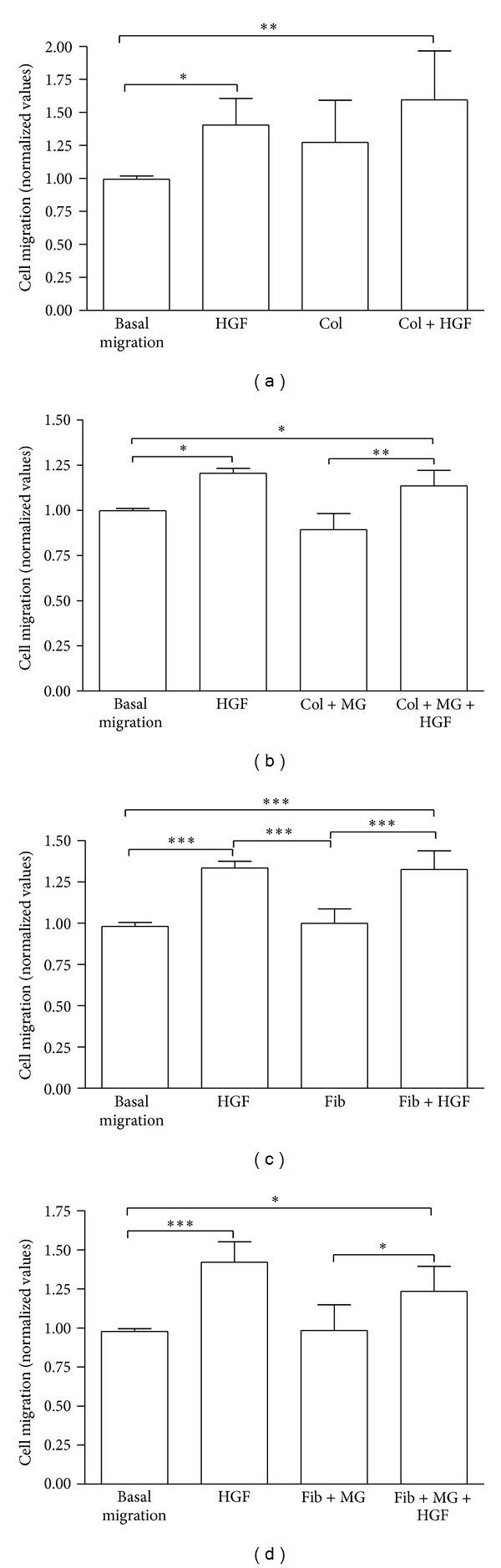
MSC migration towards biomaterials ±75 ng/mL HGF. Migration assay ±HGF in (a) collagen and (c) fibrin. Invasion assay ±HGF + layer of Matrigel on (b) collagen and (d) fibrin. All values normalized to basal migration. *P < 0.05, **P < 0.01, ***P < 0.001. All results based on *n* = 5.

HGF in medium alone (no biomaterial) showed a significant increase in cell migration compared to basal migration (without HGF, without biomaterial) as previously described for different cell types [8, 36, 42]. In addition, HGF incorporated into collagen and fibrin showed a significantly higher cell migration than basal migration (approx. 56% and 31%, resp.; Figures 8(a) and 8(c)). Since our project aims for a release of HGF with the help of wound dressings for chronic diseases or burns from a natural carrier material, these results are useful for future *in vivo* studies.

A haptotactic effect of fibrin on the migration of vascular smooth muscle cells was described early [43]. Another study on the dose-dependent effect of fibrin on bovine endothelial cells showed the positive influence of fibrin on the migratory activity of the cells [44]. In our study, we did not observe this effect of fibrin without HGF on the MSC, but only in combination with HGF (Figure 8(c)). We think that MSC might not be as susceptible to fibrin and its components as vascular and/or endothelial cells since both of these cell types were shown to play a key role in the regulation of the inflammation site in wound healing (through their interaction with fibrinogen metabolites and other migrating cells) [45]. A study combining HGF into fibrin was carried out by Zhang and colleagues, who used fibrin as an injectable biomatrix. They stated the efficacy of a PEGylated fibrin biomatrix regarding stem cell transplantation for the regeneration of myocardium in a murine model [46]. The authors considered HGF loading of biomaterials a platform technology with potential applications in other biomaterial and growth factor combinations as well. Along these lines, fibrin could serve as a potential carrier of HGF in a wound dressing.

As early as 1981, different concentrations of human collagen (type I, III, V) were found to have chemotactic effects on various types of tumor cells [47]. Later on, the chemo- and haptotactic effects of type I collagen were described for rabbit and human MSC [20]. These descriptions are in accordance with our findings. A modified assay (membrane coated with type I collagen) to evaluate the invasion of endometrial adenocarcinoma cell lines stimulated by the addition of HGF in a dose-dependent manner [48] underlines our results. Although the modification was different to ours, type I collagen was found to enhance cell migration. A few years ago, Bhargava and coworkers showed the diffusion of HGF from a collagen gel, enhancing the repair of meniscal injuries in a dog study. Particularly in combination with platelet-derived growth factor, HGF released from collagen exerted a significantly higher effect on the cell migration to the simulated defect than collagen gel alone [49].

*In vivo* models need to be performed to evaluate to what extend the direct incorporation of HGF into fibrin and collagen influences MSC migration. One study by Kanematsu et al. showed the release profile of HGF, among other growth factors, incorporated into a collagenous matrix which was implanted into mouse subcutis [34]. This group described collagen type I as a suitable carrier for growth factors regarding the *in vivo* release in a mouse model. Their results, based solely on *in vivo* experiments, indicate the functionality of our *in vitro* analyzed system in our upcoming mouse study.

Overall, the aforementioned studies are in accordance with our findings, so that HGF alone and in combination with collagen and fibrin acted as a promoter of higher MSC motility and migration.

*Invasion Assay*

**Figure 9:**
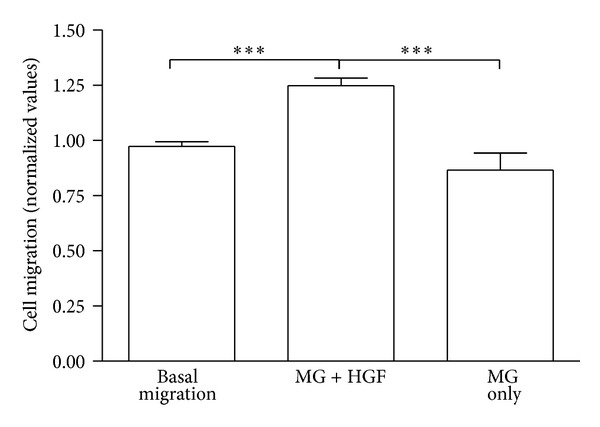
MSC invasion assay with Matrigel ±HGF. MG: Matrigel. All values normalized to basal migration. ***P < 0.001. Results represent *n* = 3.

To mimic the *in vivo* situation of foreign body response after implantation of (HGF-loaded) biomaterials, collagen and fibrin were coated with a thin layer of Matrigel. As in the migration assays, HGF in stem cell medium alone showed significantly higher cell migration than basal migration. Collagen with HGF coated with Matrigel showed a significantly higher cell migration (approx. 22%) compared to collagen without HGF but with Matrigel. The same result was found for fibrin (approx. 25%; Figures 8(b) and 8(d)). Here, Matrigel ±HGF served as control to exclude any possible chemotactic effects of Matrigel on MSC (Figure 9). We deliberately chose to coat the biomaterials on the bottom of the well (not the membrane) to check for a possible retarded release of HGF from the biomaterials in this particular experiment, which is a novel adaptation of the Boyden chamber assay, since all previous studies (exclusively in cancer research) described the coating of the membranes.

To exclude any possible chemotactic or chemokinetic effect of Matrigel on the migration of MSC, the modified Boyden chamber assay was carried out without collagen and fibrin gel, but only with Matrigel on the bottom of the plate. There is no difference in the migration of cells when the wells are coated with a thin layer of Matrigel compared to basal migration. However, when HGF is added to the bottom compartment coated with Matrigel, the cell migration is significantly enhanced (***P < 0.001, Figure 9). Therefore, we can exclude any effect of Matrigel on the cell migration and further evaluated the effect of the collagen and fibrin gels.

Even though the Boyden chamber is the method of choice for the investigation of cell motility and directed migration, studies regarding the combinatory approach of fibrin and Matrigel are absent. Matrigel-coated membrane assays have been applied in the field of angiogenesis, invasive cell migration, penetration of the basement membrane, and the preclinical development of anti-invasive and antiangiogenic agents [50–53], and they were reviewed recently [54]. However, the Boyden chamber assay has not been previously used to test growth factor-loaded biomaterials with fibrin and MSC. In an early approach with type I collagen as the major structural component of the boundary layer, the coating with type IV collagen and laminin established a selective barrier [55]. These results were similar to our findings using the combination of collagen and MSC.

When coating the Boyden chamber inserts with type IV collagen on both sides and using HGF in various concentrations (10 to 100 ng/mL) for different human lung cancer cell lines, both chemotactic and chemokinetic motilities on tumor cells were induced [56]. Another group coated both sides of the filter with type I collagen because they previously found that this was an appropriate molecule to support adhesion and migration of MSC. In their comprehensive analysis of chemotactic factors for bone marrow MSC and rabbit MSC, they concluded HGF to be an essential concentration-dependent chemoattractant for rabbit and human MSC [57]. Both studies once more emphasized the important influence of HGF on the chemotactic and chemokinetic motility of different cell types. All in all, our approach opens up new insights into the topic of growth factor-induced chemotaxis. In summary, collagen and fibrin are promising candidates as HGF carriers, which we will elucidate in further *in vivo* studies.

## 4. Conclusion

We were able to show the gradual release of HGF from both collagen and fibrin gels, which is an important prerequisite for our MSC recruitment system. Furthermore, we showed the positive effect of HGF dissolved in medium or released from fibrin or collagen gels (coated with or without Matrigel) on the directed migration and recruitment of human mesenchymal stem cells *in vitro*. Since Matrigel alone did not have any chemotactic or chemokinetic effect on the migration of the cells, we deduce enhanced migration solely from HGF and HGF in combination with fibrin and collagen gels.

In the clinics, HGF-loaded biomaterials could be immediately implanted into chronic wounds or burn areas, thus overcoming the critical issues of time and costs for *in vitro* cell expansion. To further elucidate the direct mechanisms of fibrin and collagen gels with HGF on cell migration and wound healing, we will hereafter start an *in vivo* model.

## Supplementary Material

Figure 1: The scratch assay is shown in low magnification to visualize a homogeneous migration of MSC throughout the whole scratch area.
Figure 2: The scratch assay is shown for all donors (*n*=4) to exclude donor variations in MSC migration.
