# Supplementary material for: Hepatocyte Growth Factor-Loaded Biomaterials for Mesenchymal Stem Cell Recruitment
Source: Stem Cells Int. 2013 Jun 18;2013:892065. doi: 10.1155/2013/892065 (PMC3703903; doi:10.1155/2013/892065)
Supplement: Supplementary file 1 — Figure 1: The scratch assay is shown in low magnification to visualize a homogeneous migration of MSC throughout the whole scratch area. Figure 2: The scratch assay is shown for all donors (n=4) to exclude donor variations in MSC migration. [file 892065.f1.docx]

Supplementary figure 1:


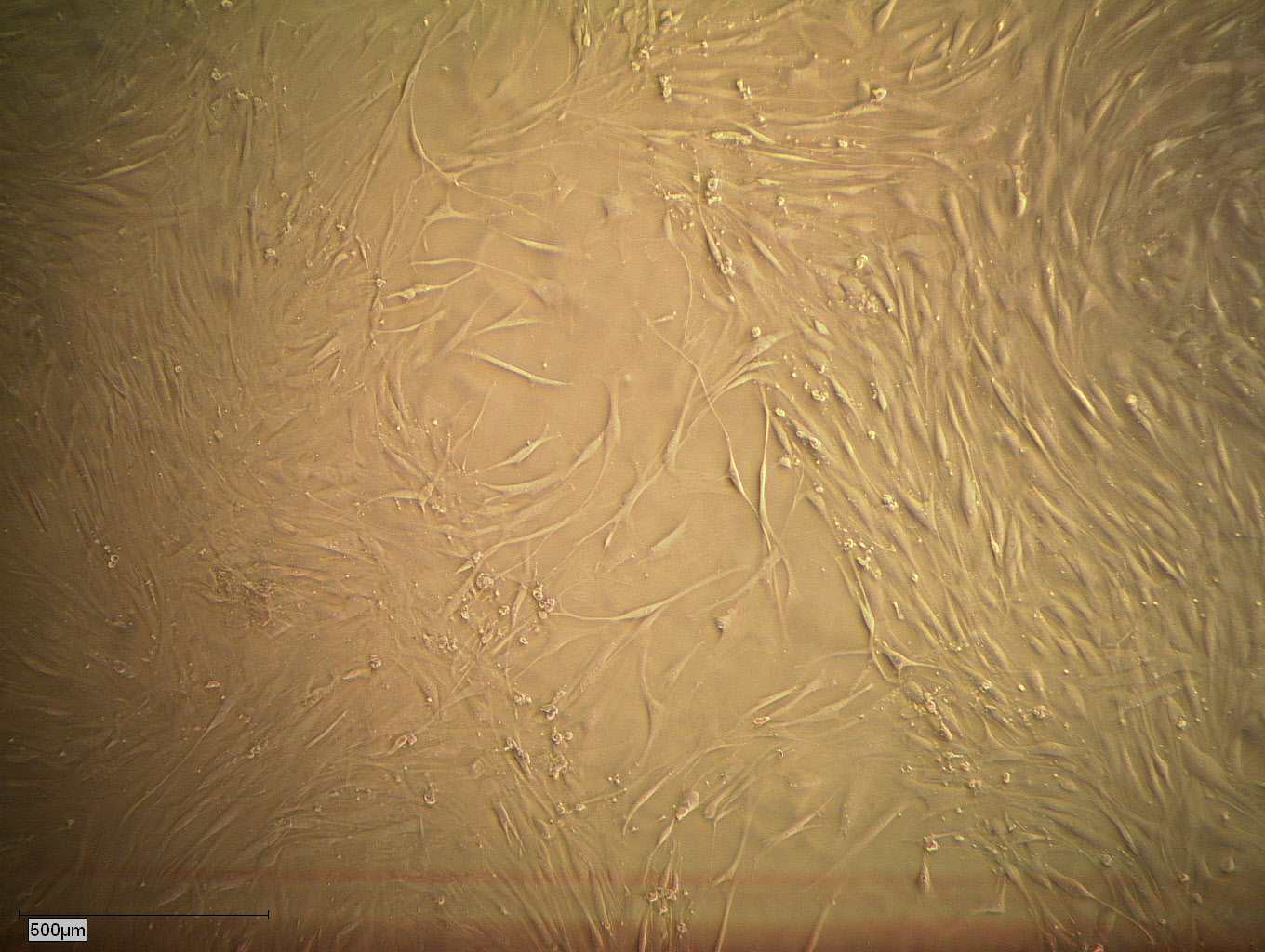


24h +HGF


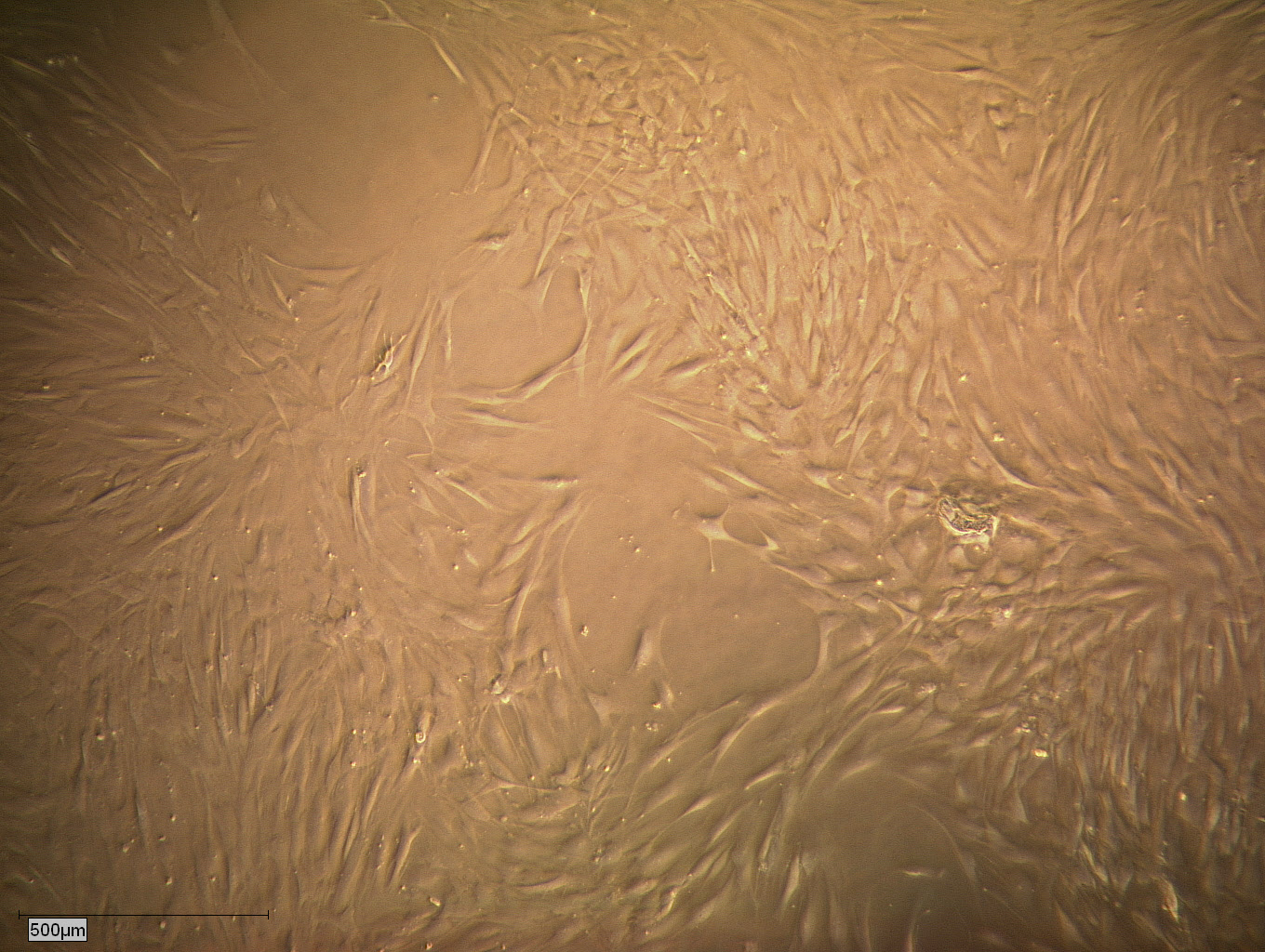


24h -HGF


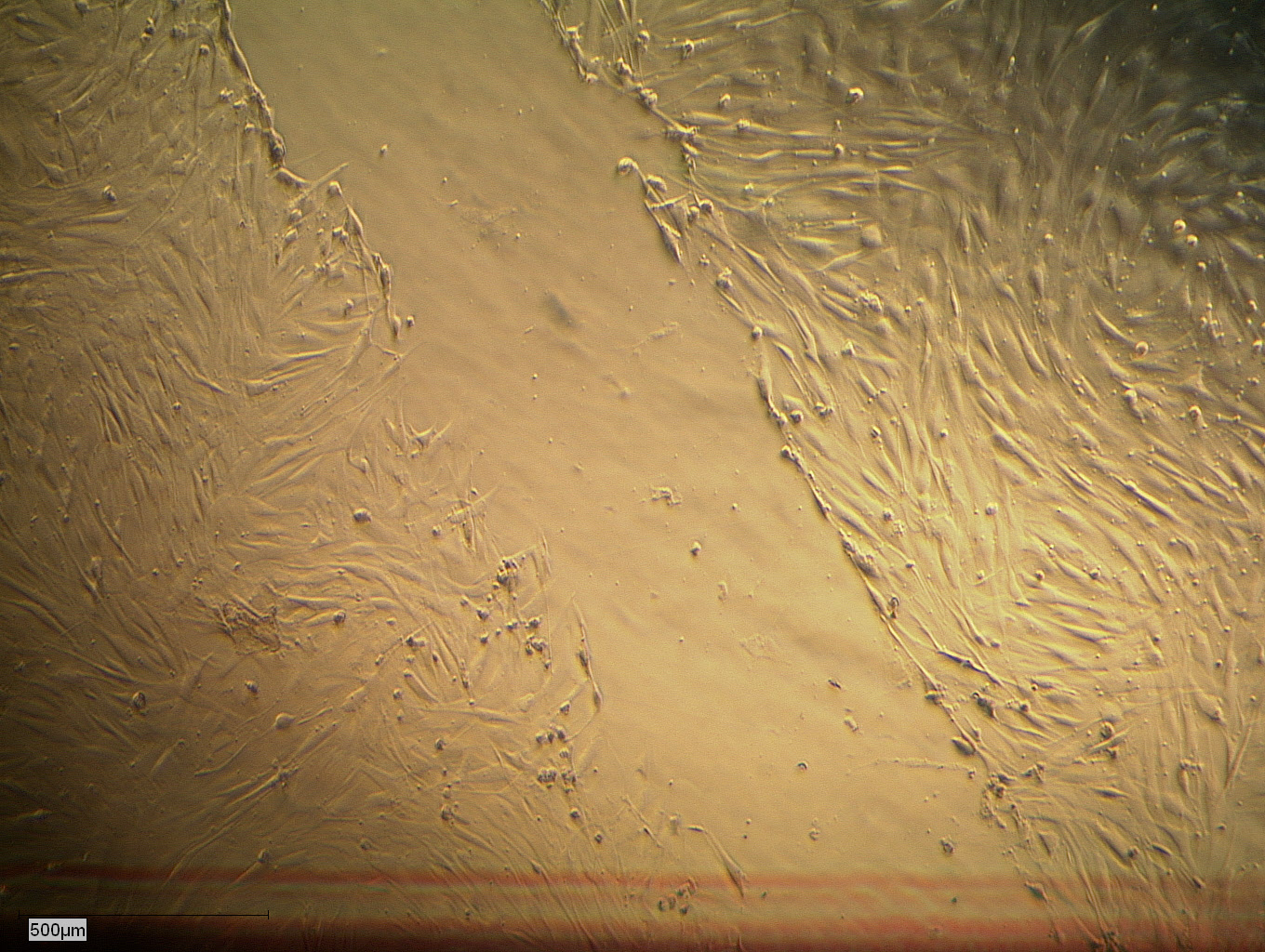


0h

Scratch Assay at low magnification for one donor after 0 h and 24 h without (-) and with (+) HGF. Scale bar = 500 µm.
