# Supplementary material for: Hepatocyte Growth Factor-Loaded Biomaterials for Mesenchymal Stem Cell Recruitment
Source: Stem Cells Int. 2013 Jun 18;2013:892065. doi: 10.1155/2013/892065 (PMC3703903; doi:10.1155/2013/892065)
Supplement: Supplementary file 2 [file 892065.f2.docx]

Supplementary figure 2:

Donor 1


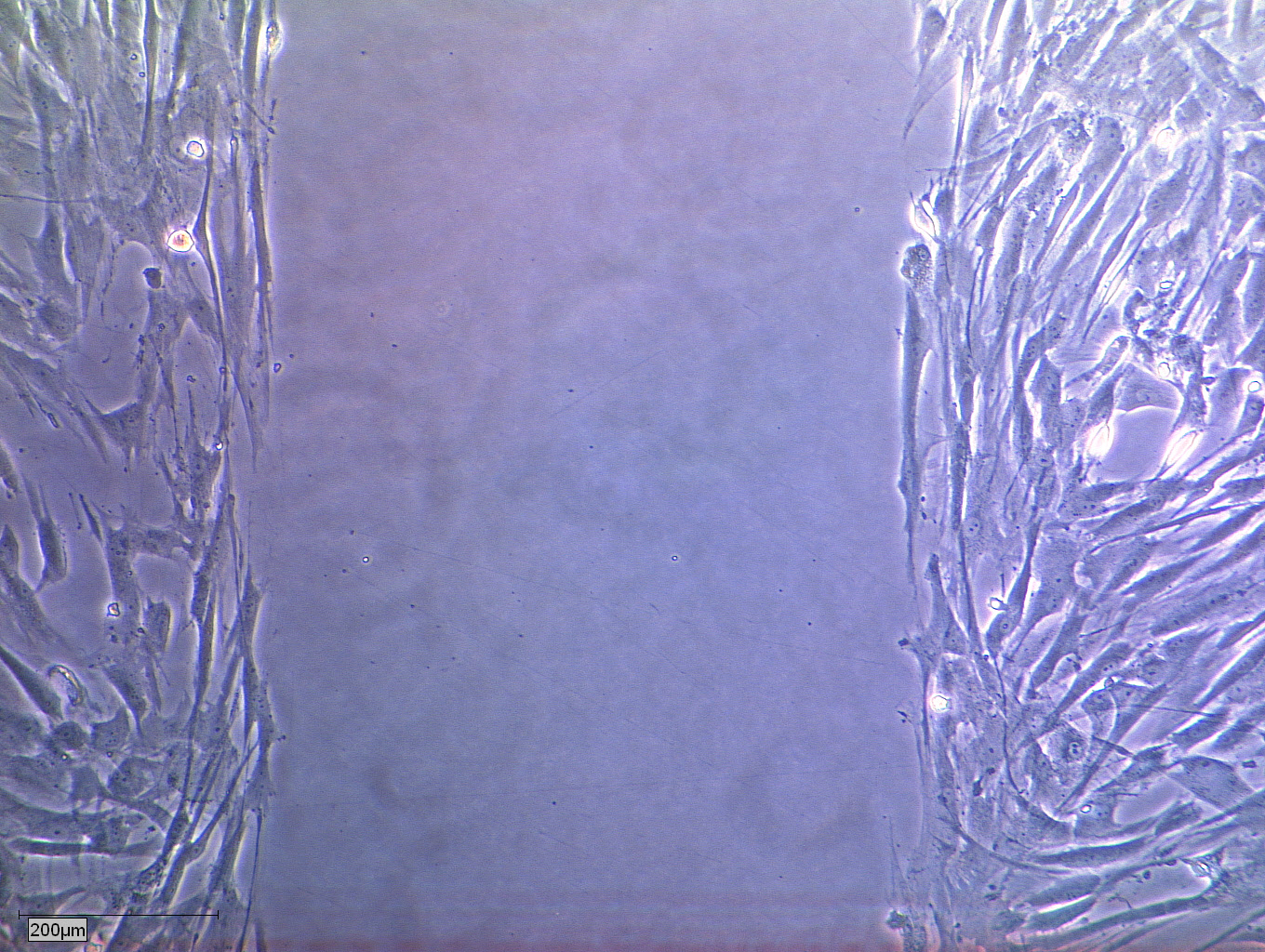

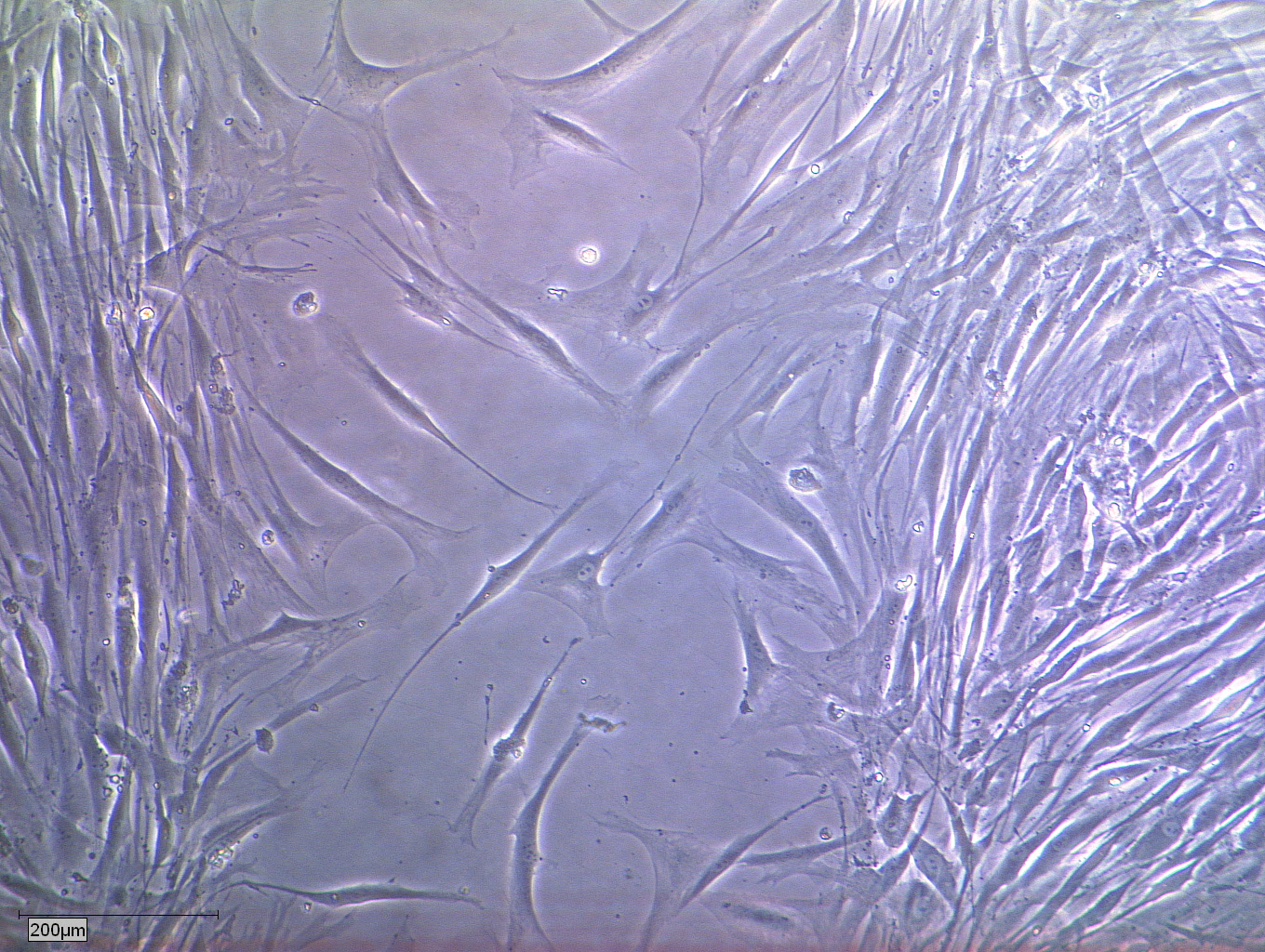

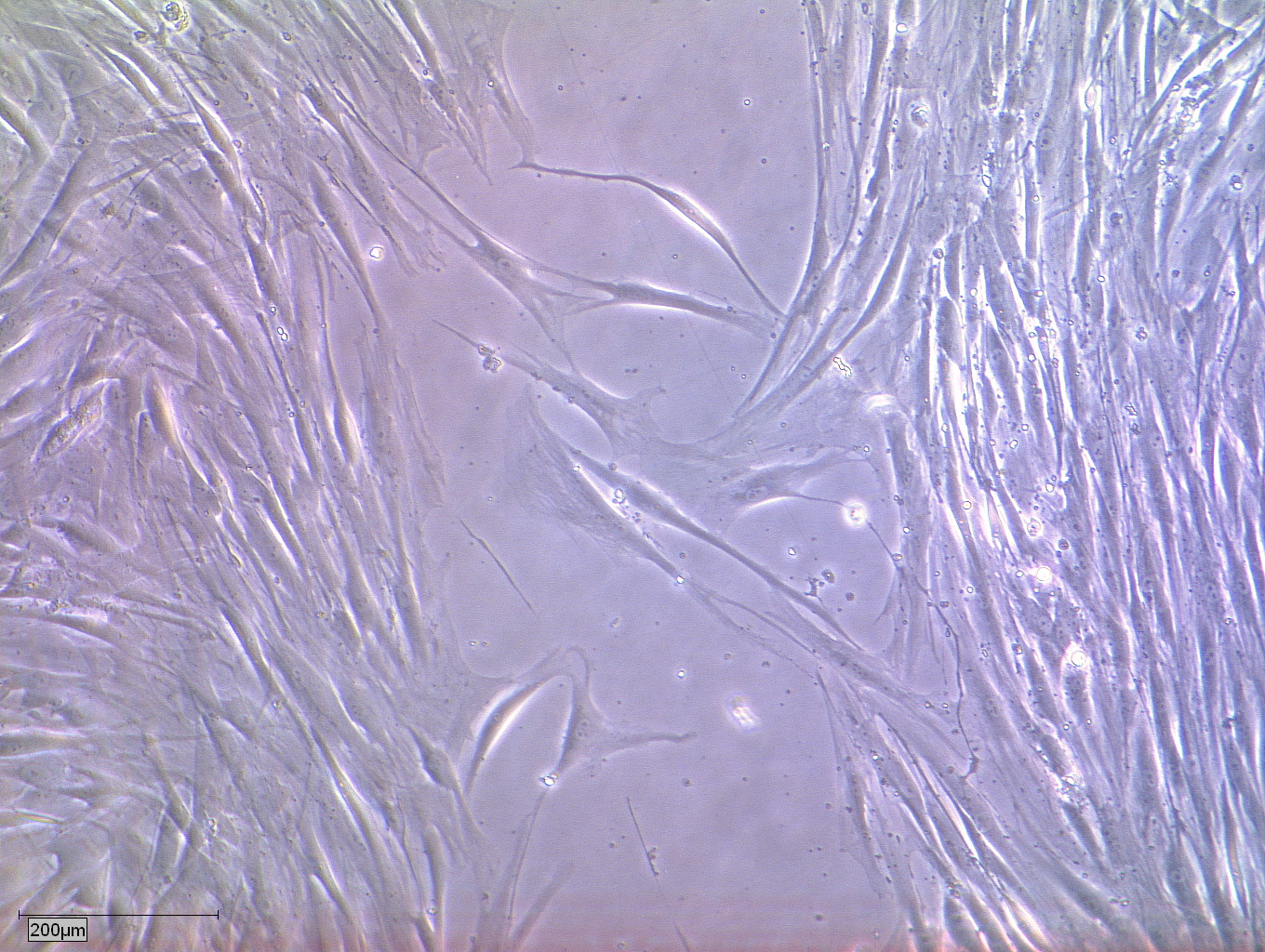


0 h

24 h -HGF

24 h +HGF


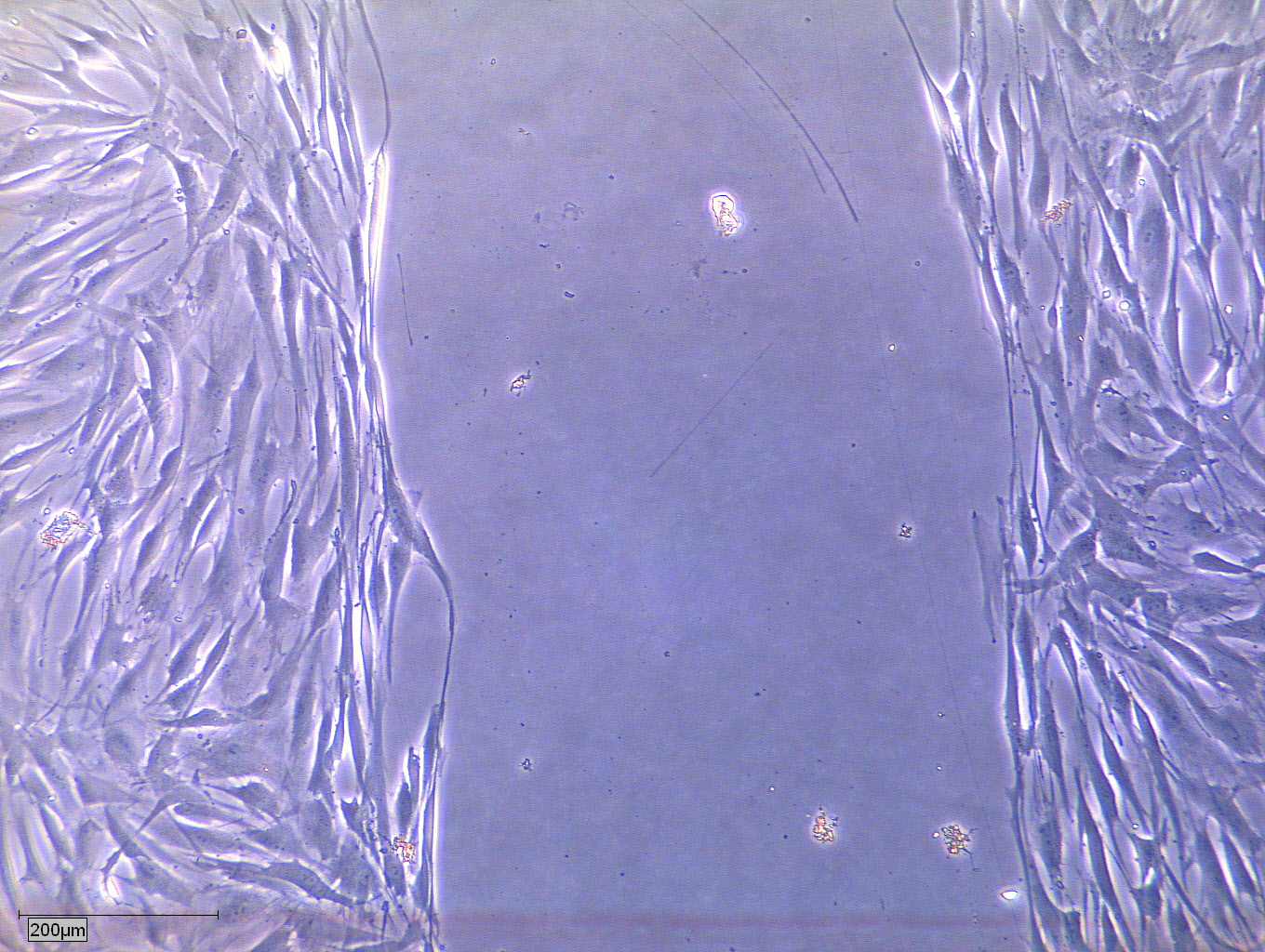

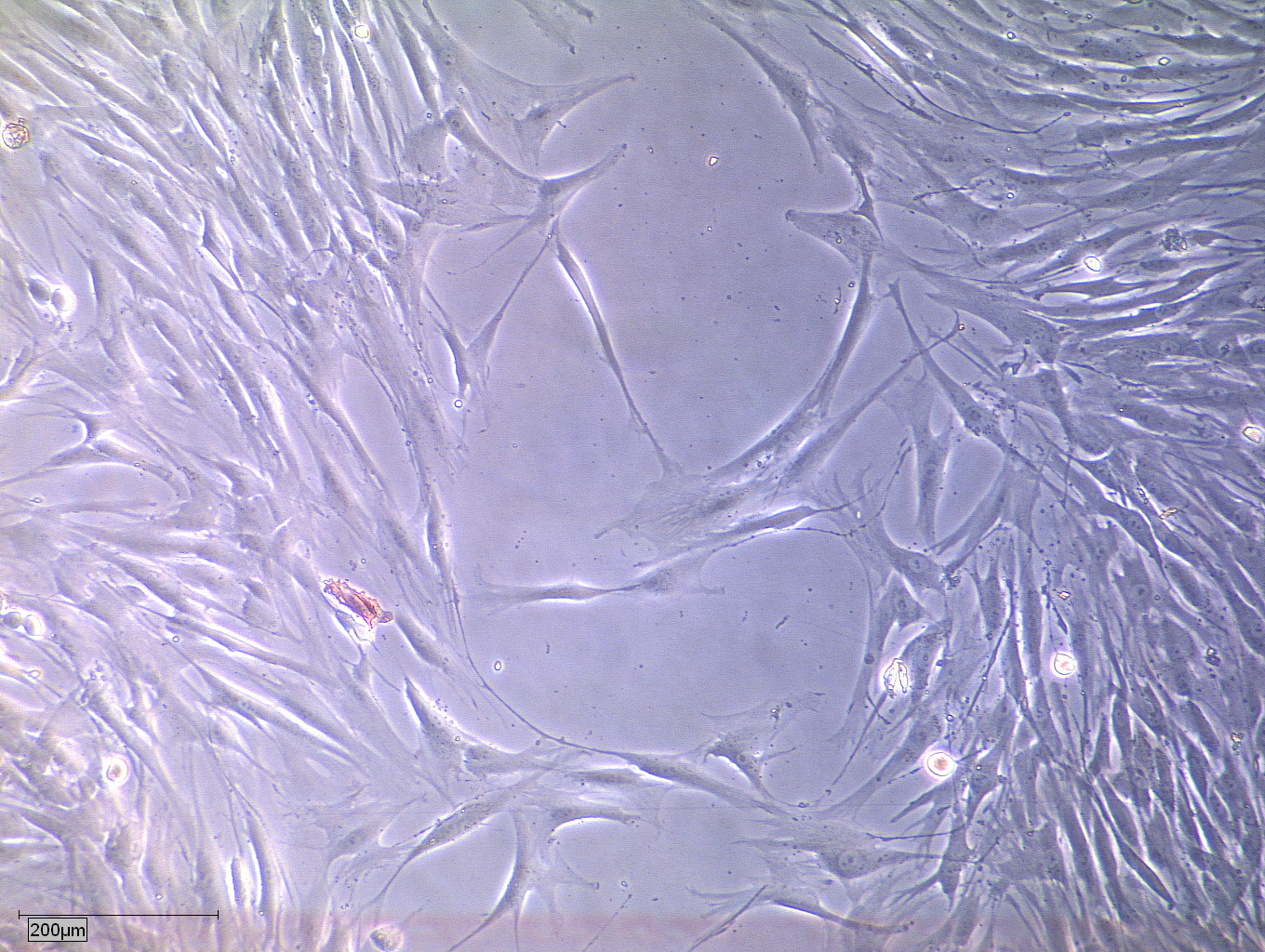

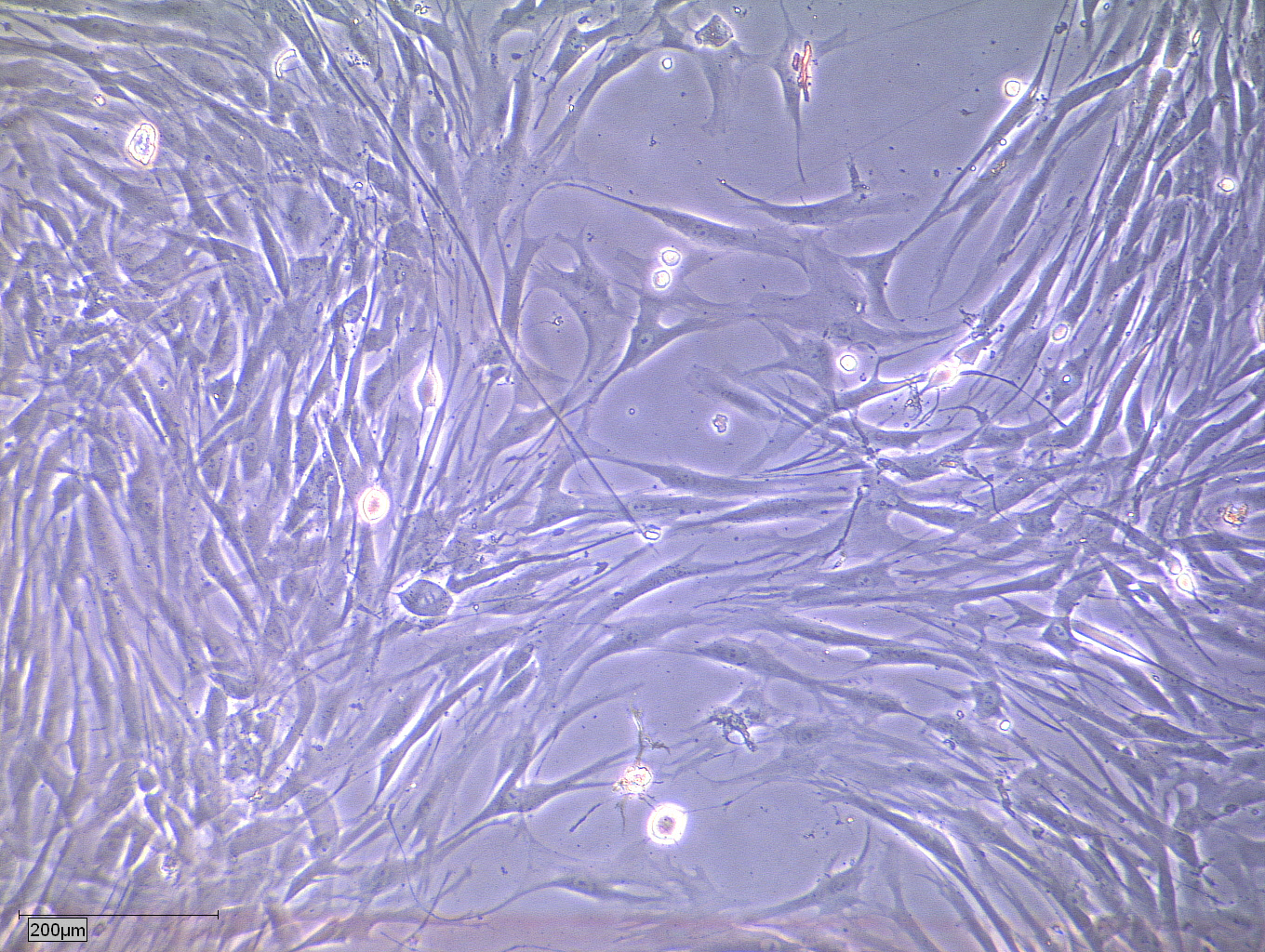


0 h

24 h -HGF

24 h +HGF


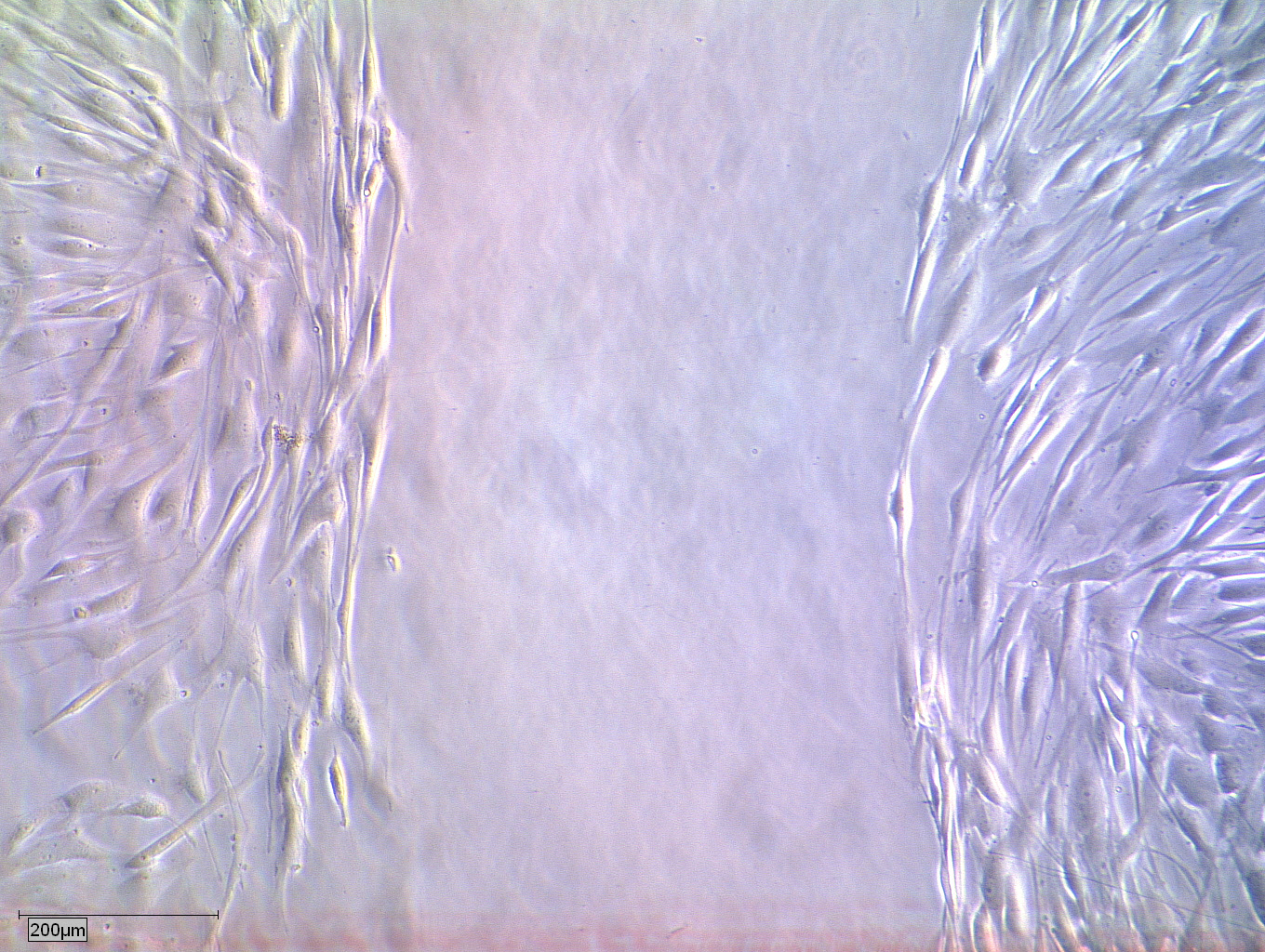

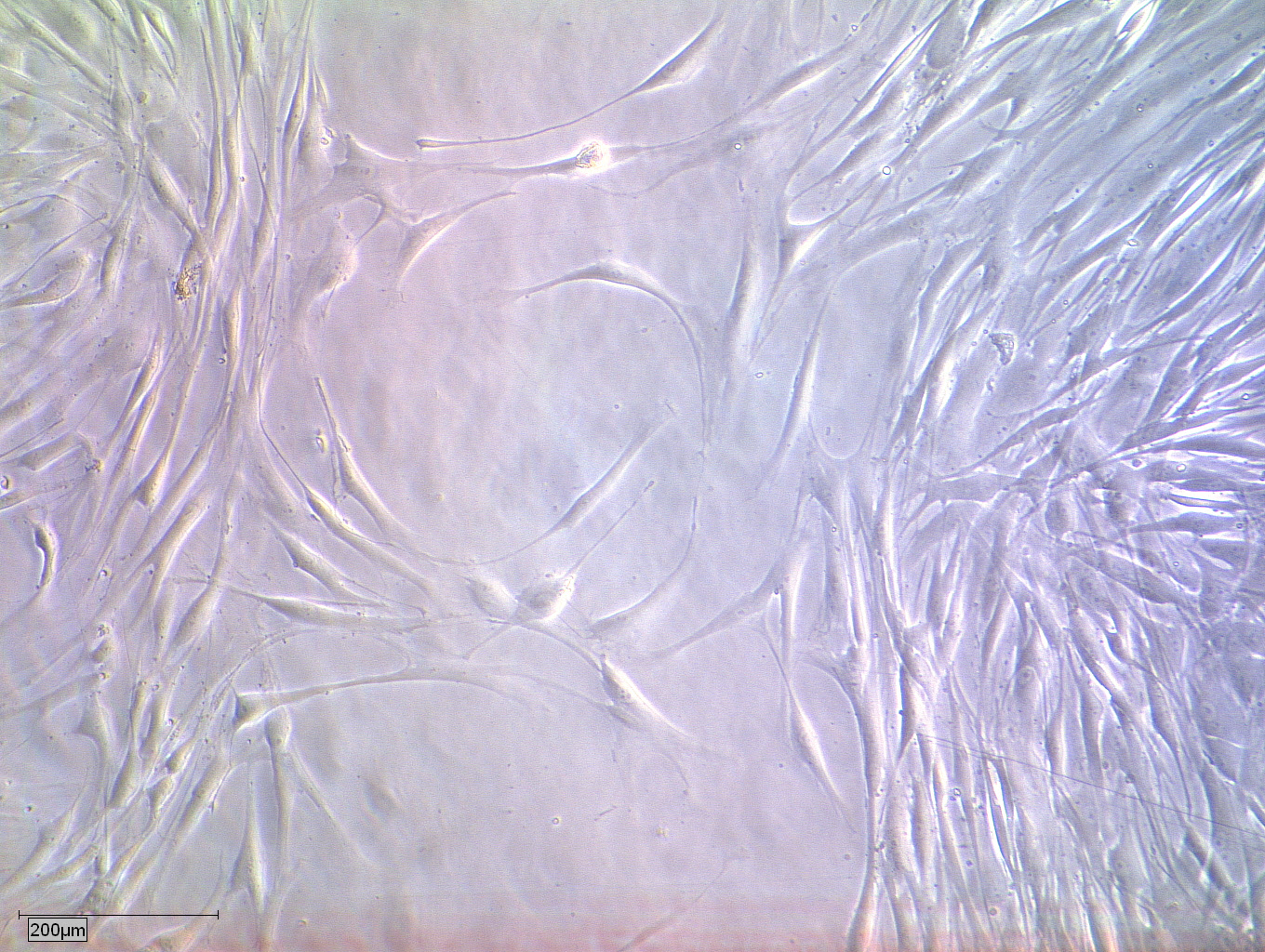

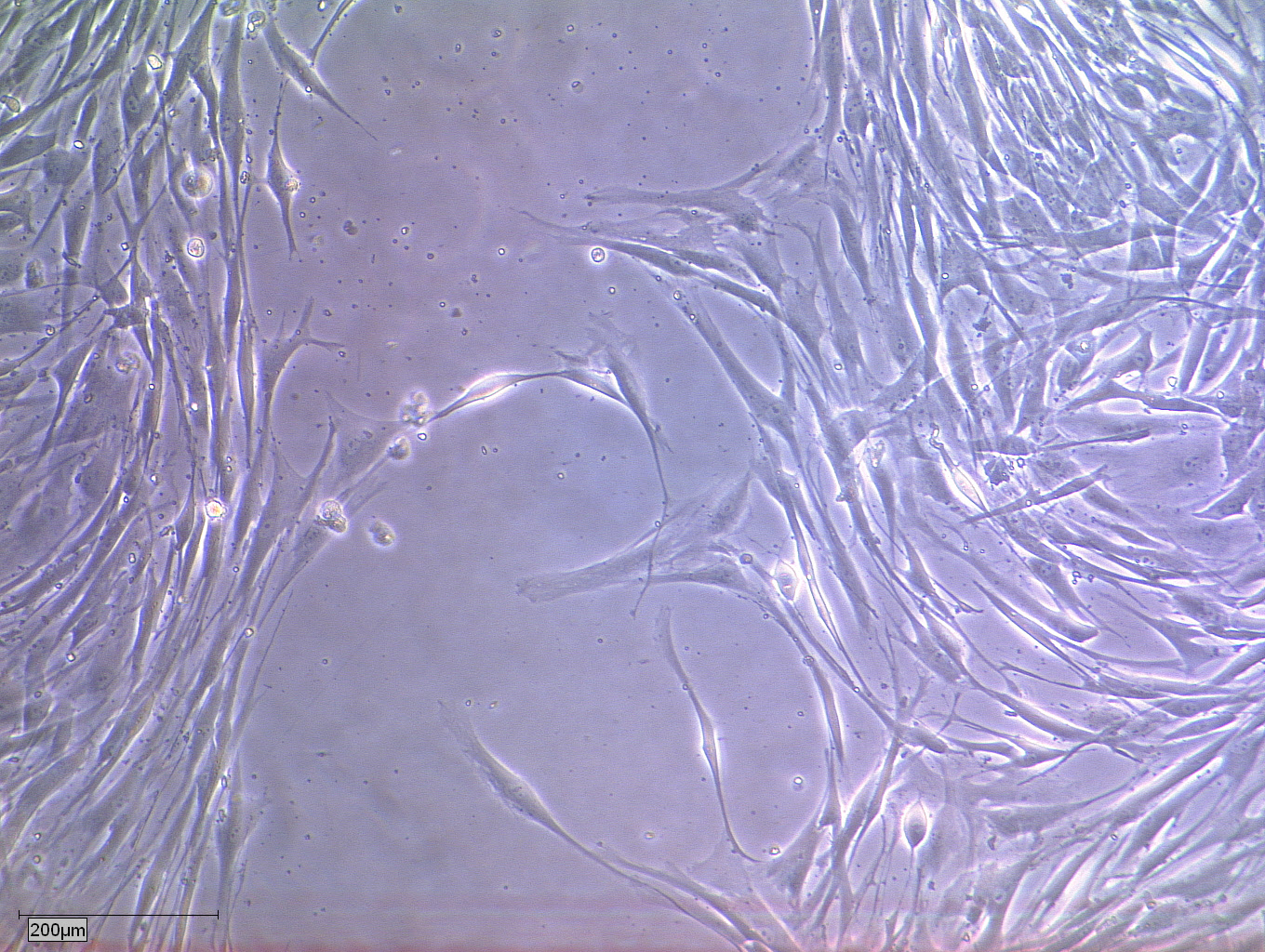


0 h

24 h -HGF

24 h +HGF


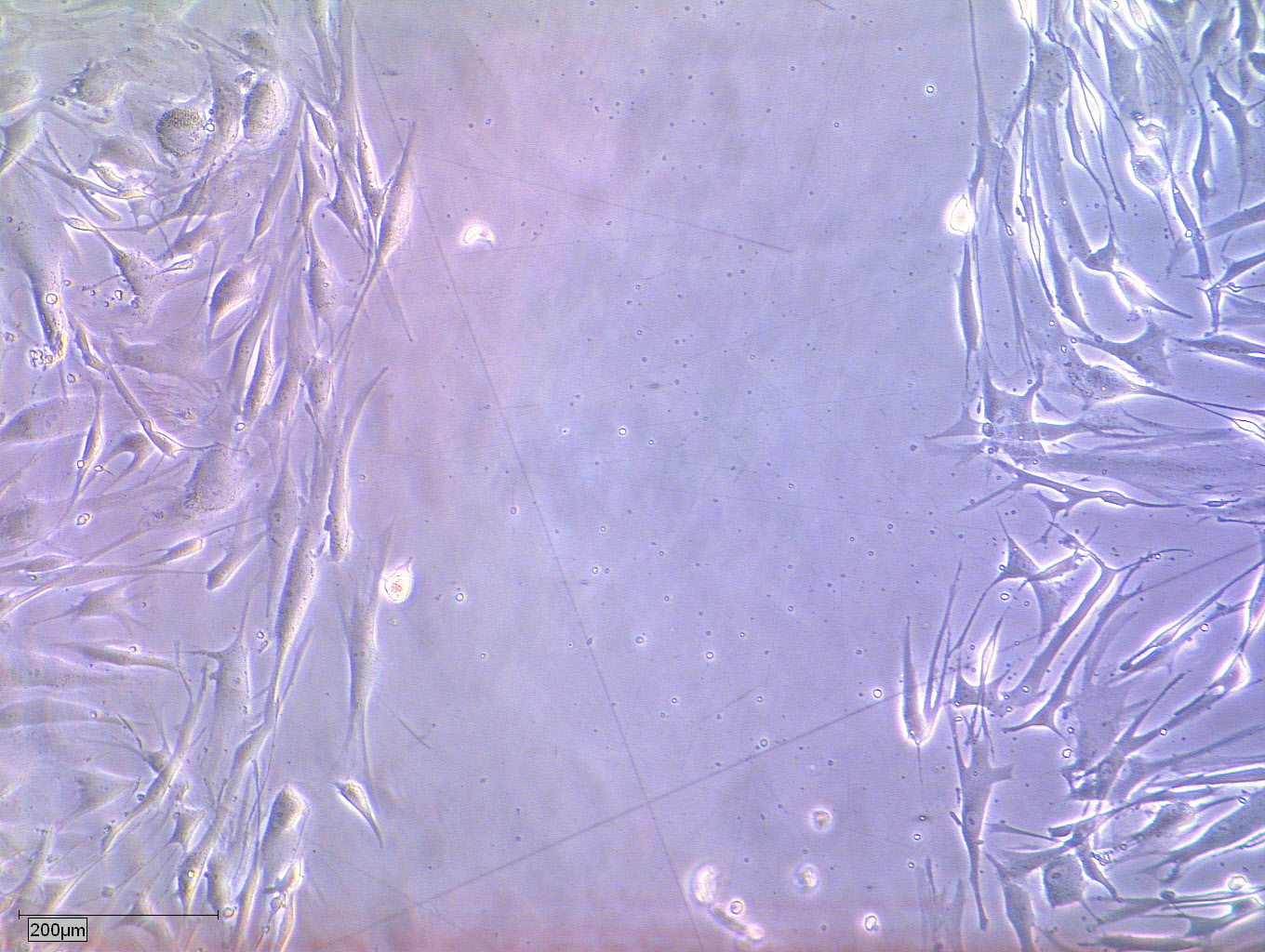

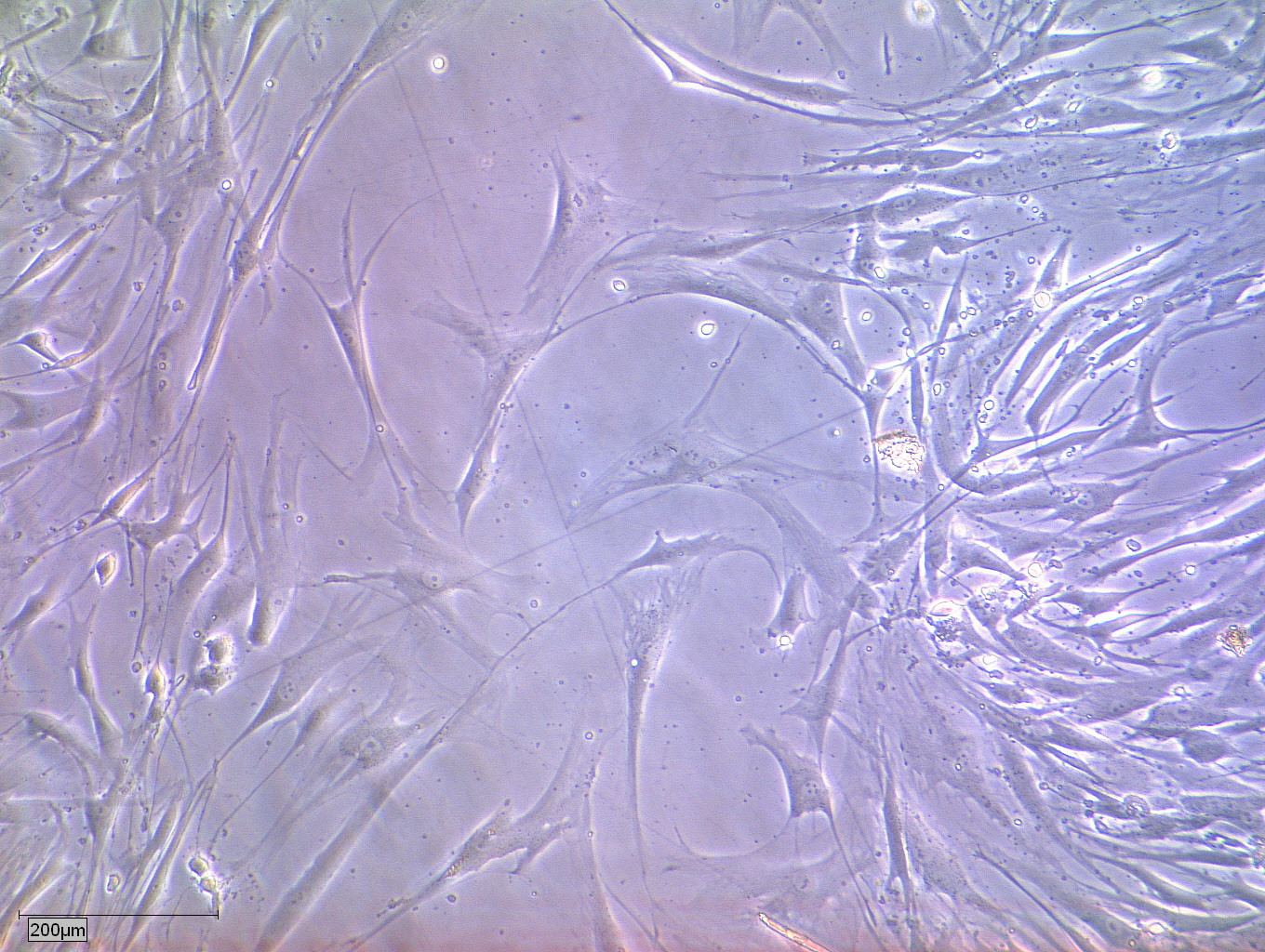

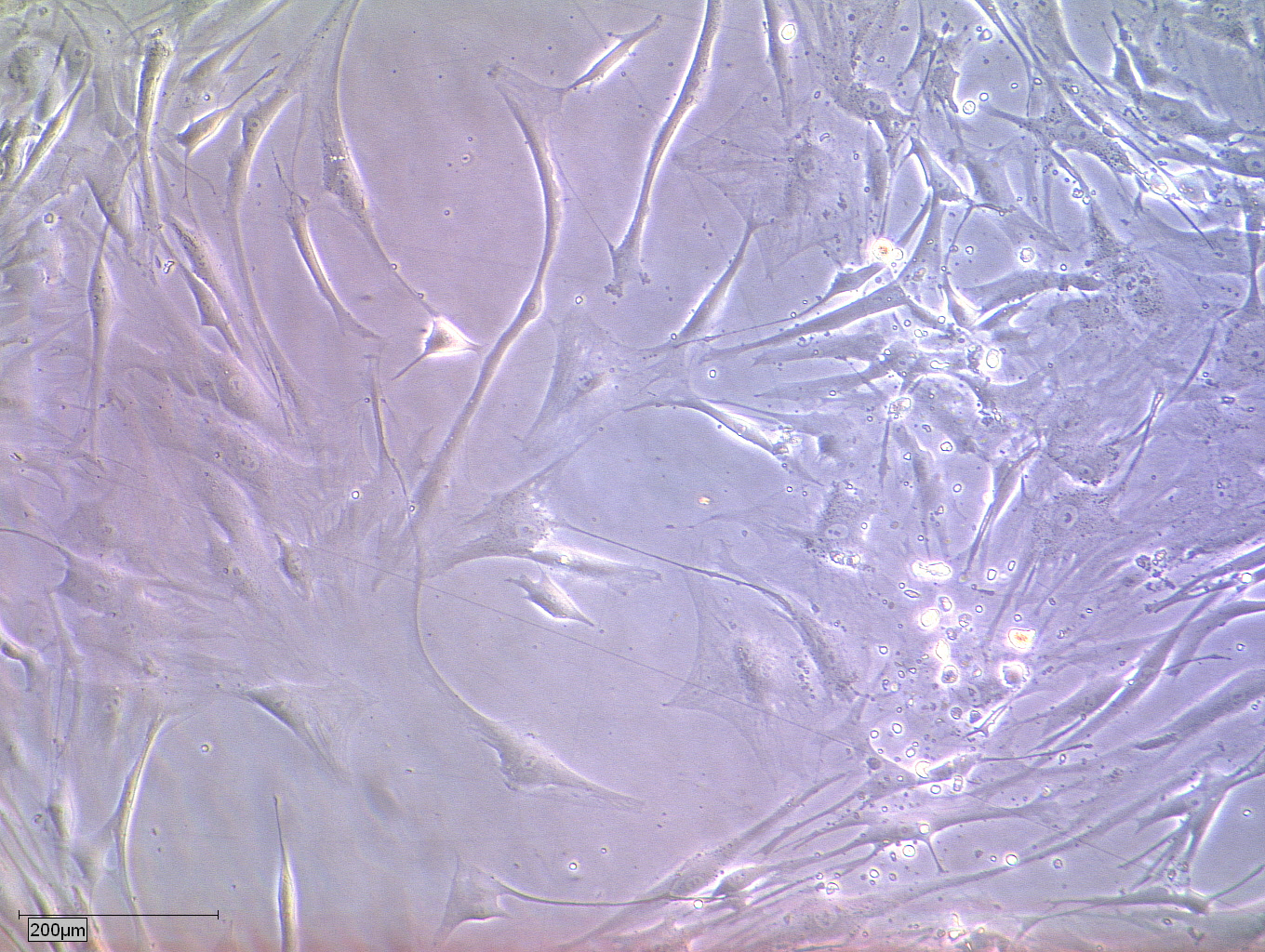


0 h

24 h -HGF

24 h +HGF

Donor 2

Donor 3

Donor 4

Scratch assay for all donors (n=4). Scale bars = 200 µm.
